# Tumor Targeting with Methotrexate-Conjugated Zwitterionic Near-Infrared Fluorophore for Precise Photothermal Therapy

**DOI:** 10.3390/ijms232214127

**Published:** 2022-11-16

**Authors:** Gayoung Jo, Eun Jeong Kim, Min Ho Park, Hoon Hyun

**Affiliations:** 1 Department of Biomedical Sciences, Chonnam National University Medical School, Hwasun 58128, Republic of Korea; 2 Department of Surgery, Chonnam National University Medical School, Hwasun 58128, Republic of Korea; 3 BioMedical Sciences Graduate Program (BMSGP), Chonnam National University, Hwasun 58128, Republic of Korea

**Keywords:** methotrexate, zwitterionic fluorophores, near-infrared fluorescence imaging, photothermal therapy, tumor targeting

## Abstract

Targeted tumor imaging can effectively enable image-guided surgery and precise cancer therapy. Finding the right combination of anticancer drugs and near-infrared (NIR) fluorophores is the key to targeted photothermal cancer treatment. In this study, a tumor-targetable NIR fluorophore conjugate with rapid body clearance was developed for accurate tumor imaging and effective photothermal therapy (PTT). The methotrexate (MTX) and zwitterionic NIR fluorophore conjugate (MTX-ZW) were prepared by conjugating a folate antagonist MTX with an aminated ZW800-1 analog to increase the tumor targetability for NIR laser-based PTT of cancer. The MTX, known as a poor tumor-selective drug, showed high tumor accumulation and rapid background clearance after conjugation with the highly water-soluble zwitterionic NIR fluorophore up to 4 h post-injection. The photothermal energy was generated from the MTX-ZW conjugate to induce necrotic cell death in the targeted tumor site under 808 nm laser irradiation. Compared with the previously reported MTX conjugates, the MTX-ZW conjugate can be a great candidate for targeted tumor imaging and fluorescence-guided photothermal cancer therapy. Therefore, these results provide a strategy for the design of drug-fluorophore conjugates and elaborate therapeutic platforms for cancer phototherapy.

## 1. Introduction

Recently, the near-infrared (NIR) fluorophore-based photothermal therapy (PTT) has gained significant attraction in the field of cancer treatments because of the NIR fluorescence imaging guidance, high photothermal conversion efficiency, and strong NIR light absorption of the NIR fluorophore in deep biological tissues [1,2,3]. The right selection of NIR fluorophores is key to improving targeted tumor imaging and cancer phototherapy. To enhance the tumor accumulation of NIR fluorophores, a series of NIR fluorophores can be conjugated with various tumor-targeting ligands and anticancer drugs to recognize tumor microenvironments and overexpressed biomarkers [4,5,6,7]. However, tumor targetability and in vivo performance of the conjugates remain challenging because the specificity of ligands or drugs can also be reduced by the physicochemical properties of the conjugated NIR fluorophores [2,7]. In this regard, highly engineered nanomaterials have been developed for biomedical applications, including bioimaging, drug delivery, and NIR-based phototherapy, due to their superior optical characteristics [8,9,10].

Despite the significant progress in the NIR fluorophore-based PTT of cancer, most NIR fluorophores are still unavailable in clinic, except for indocyanine green (ICG), which is clinically available but not chemically conjugatable, due to the unsolved biological safety issues such as cytotoxicity, nonspecific tissue/organ uptake, and delayed excretion [11]. Previously, commercially available heptamethine cyanine fluorophores, including IR-780, IR-783, IR-786, and IR-808 (also called MHI-148), have been typically used for targeted cancer phototherapy after conjugation with various ligands or drugs [12]. Owing to their high hydrophobicity and cytotoxicity, however, the water-insoluble anticancer drugs and small-molecule ligands are mostly difficult to use with commercial NIR fluorophores after conjugation. Thus, the development of more hydrophilic and less cytotoxic NIR fluorophores has been required for the conjugation with hydrophobic anticancer drugs to use in targeted cancer phototherapy.

To overcome the limitations, a highly water-soluble zwitterionic NIR fluorophore, named ZW800-1, was initially developed by Choi et al. showing excellent optical properties, including high molar extinction coefficient, photothermal conversion efficiency, and good water solubility compared to the clinically available ICG and other conventional NIR fluorophores [11,13]. Additionally, ZW800-1 has remarkable advantages for in vivo NIR fluorescence imaging, owing to ultralow nonspecific tissue/organ uptake and rapid renal excretion from the body, so that it can provide a higher signal-to-background ratio after conjugation with various small-molecule ligands [4,7]. Based on the improved in vivo performance and photothermal property of ZW800-1, the hydrophobic anticancer drugs and small-molecule ligands can be utilized after conjugation with the ZW800-1 for targeted tumor imaging and effective photothermal cancer treatment.

Methotrexate (MTX), a structural analog of folic acid, shows therapeutic effects on many different kinds of cancer cells that overexpress folate receptors on the cell membrane surface [14]. Although the folate antagonist MTX is widely used as an antiproliferative agent in cancer treatments, the use of MTX is still limited due to its systemic toxicity and adverse effects, as well as its lack of tumor selectivity [15,16]. Additionally, MTX possesses low cellular permeability (C log*P* = 0.53), poor aqueous solubility (0.01 mg/mL), and short plasma *t*_1/2_ (2–10 h), resulting in low bioavailability and pharmacokinetics [17,18]. Previously, conventional methods using many types of nanomaterials as a carrier of MTX have been mainly reported to improve the therapeutic effect of MTX [19,20,21,22].

**Scheme 1:**
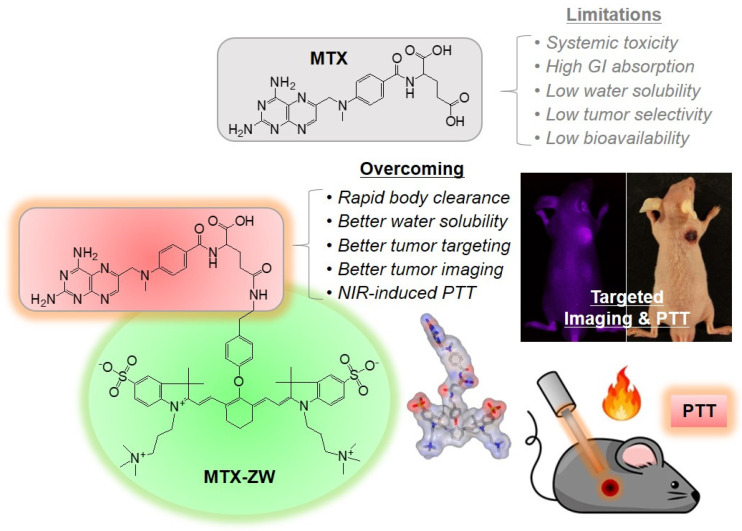
Schematic representation of the MTX-ZW conjugate for tumor-targeted imaging and PTT. The right combination of the poorly water-soluble MTX and highly water-soluble zwitterionic NIR fluorophore is the key to overcoming the limitations of MTX for improved cancer phototherapy.

The combination of poorly water-soluble MTX with highly water-soluble zwitterionic NIR fluorophore is a simple and effective approach to address those limitations of MTX (Scheme 1). In this study, we developed a tumor-targeted PTT agent by conjugating the antineoplastic drug MTX with the zwitterionic NIR fluorophore after the tyramine linker substitution for amide bond formation, named MTX-ZW, to improve the in vivo performance and fluorescence-guided cancer PTT. To the best of our knowledge, no study has investigated the effectiveness of the MTX-ZW conjugate in cancer PTT application. Therefore, this study provides a promising strategy for the development of tumor-targeted drug conjugates by employing the zwitterionic NIR fluorophore to enable safe and effective preclinical PTT applications.

## 2. Results

### 2.1. Synthesis and Characterization of MTX-ZW Conjugate

**Figure 1:**
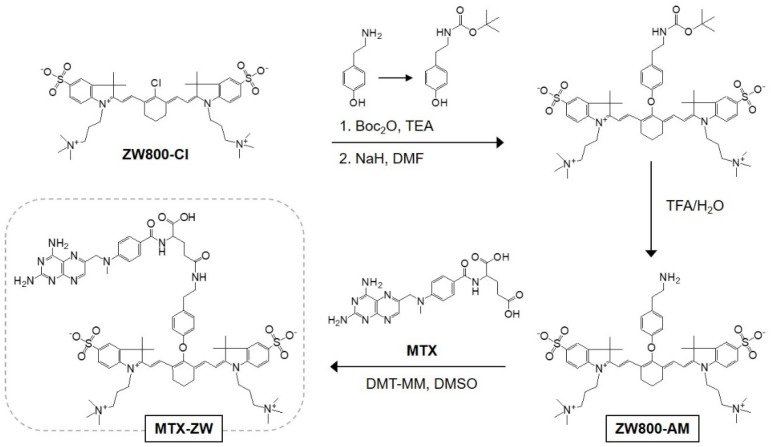
Synthetic scheme of ZW800-AM and MTX-ZW conjugate. The amine-functionalized zwitterionic NIR fluorophore ZW800-AM was prepared using the chloro-substituted ZW800-Cl via a nucleophilic displacement reaction of Boc-protected tyramine. After Boc deprotection, ZW800-AM can be covalently conjugated with MTX by forming the amide bond in a condensation reaction.

Before conjugation with the carboxylate moiety of MTX, the original structure of ZW800-1 was modified with the amine-functionalized structure of ZW800-AM, which was previously developed by Njiojob et al. [23]. ZW800-AM was prepared using the chloro-substituted ZW800-Cl and amine-functionalized tyramine through a nucleophilic displacement reaction (Figure 1). To avoid an unnecessary reaction between the amine group of tyramine and the chloro-cyclohexene on the heptamethine core of ZW800-Cl, the amine group of tyramine was protected by the tert-butoxycarbonyl (Boc) group before use. By using Boc-protected tyramine, the phenolic moiety could be linked to the meso-carbon of the heptamethine bridge of ZW800-Cl because the selective reactivity of the phenoxide ion of tyramine was ensured by protection of the primary amine group to the substitution reaction with ZW800-Cl. To increase the nucleophilicity of the protonated oxygen, sodium hydride was used to generate the phenoxide ions in situ and accelerate the reaction with ZW800-Cl. Subsequently, the Boc-protected group was removed by treatment with a solution of trifluoroacetic acid (TFA) and water, and the final form of ZW800-AM can be utilized in amide bond formation with the MTX through a condensation reaction in the presence of a coupling agent, 4-(4,6-dimethoxy-1,3,5-triazin-2-yl)-4-methylmorpholinium chloride (DMT-MM). The terminal carboxyl group of MTX reacts with DMT-MM to form the active ester, releasing *N*-methylmorpholinium as a part of DMT-MM, then the active ester is highly reactive and can undergo a nucleophilic attack by the amine group of ZW800-AM. Finally, another part of DMT-MM, 4,6,-dimethoxy-1,3,5-triazin-2-ol, is released, and the corresponding amide bond of MTX-ZW is formed.

**Figure 2:**
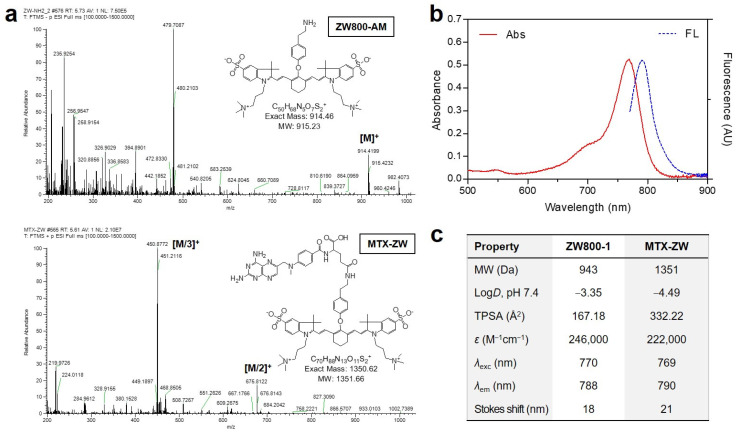
(**a**) Mass spectra of the ZW800-AM and MTX-ZW conjugate, (**b**) absorbance and fluorescence emission spectra of the MTX-ZW conjugate, (**c**) physicochemical and optical properties of the MTX-ZW conjugate compared to that of the first zwitterionic NIR fluorophore ZW800-1. Optical measurements were performed in PBS at pH 7.4. In silico calculations of the distribution coefficient (log*D* at pH 7.4) and topological polar surface area (TPSA) were performed using the Marvin and JChem calculator plugins (ChemAxon).

To confirm the successful synthesis, the exact molecular weights of the ZW800-AM and MTX-ZW conjugate were analyzed by mass spectrometry (Figure 2a). After identifying the well-defined MTX-ZW conjugate, the optical properties were measured in phosphate-buffered saline (PBS, pH 7.4). The absorption and fluorescence emission peaks of MTX-ZW exhibited in the NIR spectral region at 769 and 790 nm, respectively (Figure 2b). This suggests that the MTX-ZW conjugate can be used for the PTT application, compatible with the NIR laser system. Additionally, in silico predictions of the physicochemical properties, such as hydrophobicity (log*D*) and polarity (TPSA), were calculated using JChem (ChemAxon) (Figure 2c). The MTX-ZW conjugate displayed increased water solubility (log*D* = −4.49) and polarity (TPSA = 332.22 Å^2^), compared to that of ZW800-1 (−3.35 and 167.18 Å^2^, respectively). Thus, the improved physicochemical properties of MTX-ZW may play important roles in overcoming the limitations of MTX, such as systemic toxicity, high gastrointestinal uptake, low water solubility, and low tumor selectivity.

### 2.2. In Vitro Cytotoxicity and Cellular Uptake

**Figure 3:**
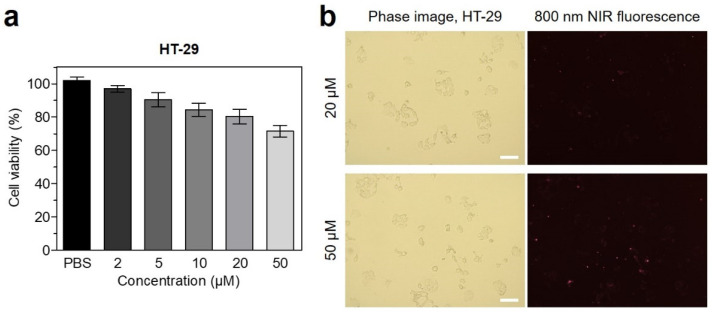
(**a**) Cell viability assay of the MTX-ZW conjugate using HT-29 cancer cells. The percentage cytotoxicity was determined after 24 h of treatment with various concentrations of the MTX-ZW conjugate. (**b**) Live cancer cell binding of the MTX-ZW conjugate in HT-29 cells 24 h after treatment with various concentrations of MTX-ZW. The phase contrast and NIR fluorescence images of the cell line were obtained using 20 and 50 µM of MTX-ZW. All NIR fluorescence images have identical exposure times and normalization. Scale bars = 100 μm.

The in vitro cell viability was investigated using the MTT assay with various concentrations of MTX-ZW in HT-29 cancer cells for 24 h (Figure 3a). The cell viability decreased gradually with increasing concentrations of MTX-ZW, which is considered to be induced by the MTX moiety of MTX-ZW because the zwitterionic NIR fluorophores such as ZW800-1 and ZW800-AM have excellent cytocompatibility as reported previously [24,25]. Moreover, the intracellular uptake of MTX-ZW was observed under NIR fluorescence microscopy after 24 h of incubation in HT-29 cancer cells (Figure 3b). Interestingly, the fluorescence signals in all conditions were very weak and slightly detected in the cancer cells treated with the high concentrations (20 and 50 μM) of MTX-ZW. Furthermore, a significant decrease in the number of viable cells was observed in the groups treated with high concentrations of MTX-ZW, which is consistent with the results of the cell viability assay. This indicates that the zwitterionic characteristic of the ZW800 moiety and the low cellular permeability of the MTX moiety in the MTX-ZW structure may contribute to reducing the binding specificity toward cancer cells. Additionally, the higher polarity (TPSA = 332.22 Å^2^) of MTX-ZW may also affect poor membrane permeability because the TPSA value greater than 140 Å^2^ tends to be less favorable binding to the cell membrane, known as the physicochemical property guidelines of Lipinski’s rule [26].

### 2.3. Time-Dependent In Vivo Tumor Imaging and Biodistribution

**Figure 4:**
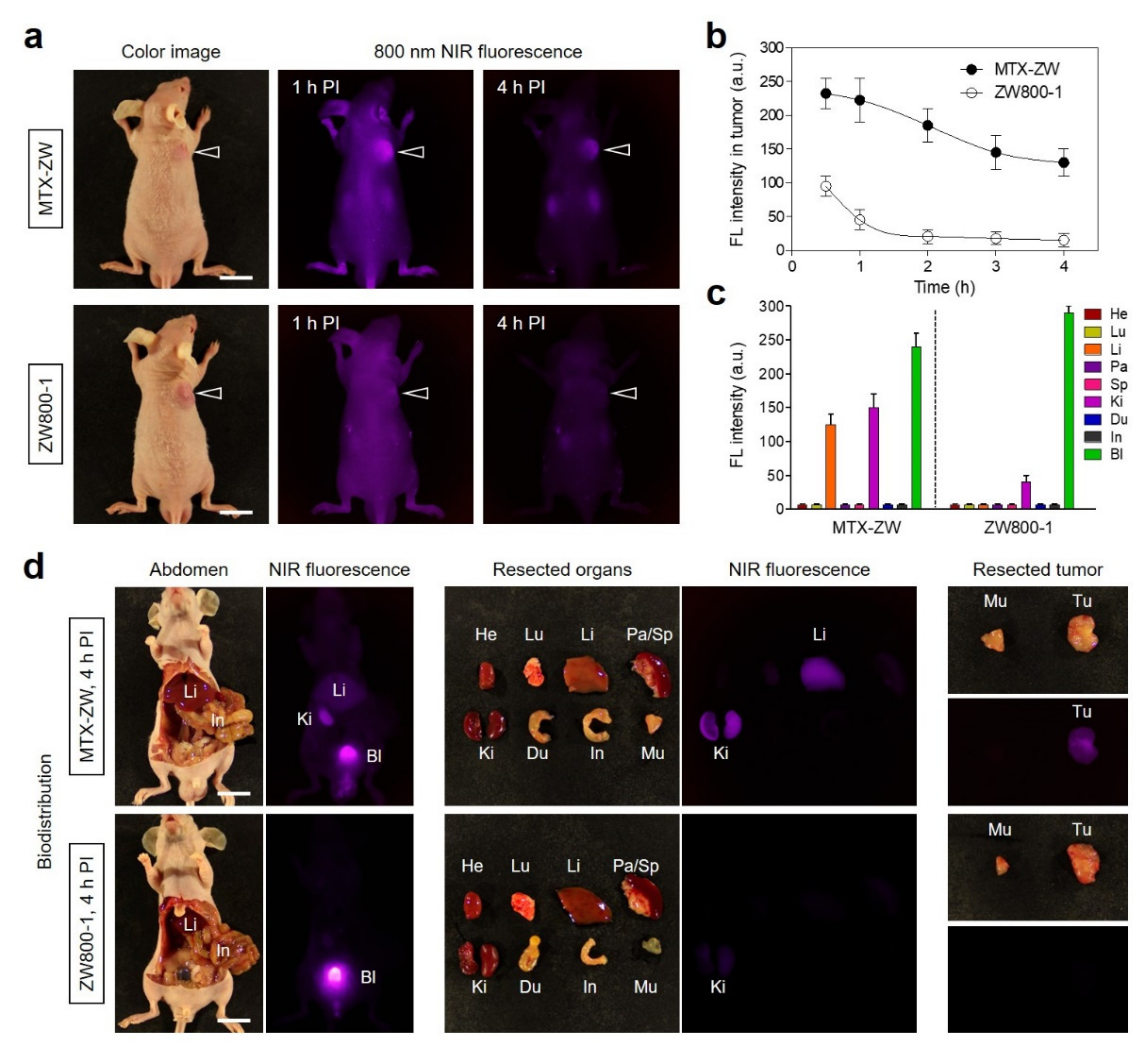
In vivo HT-29 tumor targeting efficiency and biodistribution of the MTX-ZW conjugate. (**a**) NIR fluorescence imaging 4 h after injection of MTX-ZW and ZW800-1. (**b**) Time-dependent fluorescence intensities at the tumor sites targeted by MTX-ZW and ZW800-1. (**c**) Quantitative fluorescence analysis of intraoperative dissected organs 4 h after injection of MTX-ZW and ZW800-1. (**d**) Biodistribution, resected major organs, and harvested tumor tissue imaged 4 h after injection of MTX-ZW and ZW800-1. The tumor sites are indicated by arrowheads. Abbreviations: Bl, bladder; Du, duodenum; He, heart; In, intestines; Ki, kidneys; Li, liver; Lu, lungs; Mu, muscle; Pa, pancreas; Sp, spleen; Tu, tumor; PI, post-injection. Scale bars = 1 cm. Images are representative of 3 mice per treatment group. All NIR fluorescence images had identical exposure times and normalization.

The tumor-specific targeting and biodistribution of MTX-ZW in vivo were investigated using an HT-29 xenograft mouse model to determine whether it is applicable for PTT. MTX-ZW or ZW800-1 as a control group were intravenously injected into the tumor-bearing mice. Subsequently, the mice in each group were imaged for 4 h using a real-time NIR fluorescence imaging system (Figure 4a). The mice treated with MTX-ZW revealed rapid tumor accumulation at 1 h post-injection, while the original zwitterionic NIR fluorophore ZW800-1 showed no tumor-specific uptake within 4 h after injection, corresponding to the previous reports [11,13]. The fluorescence intensity at the tumor tissue treated with MTX-ZW gradually decreased within 4 h after injection, and the fluorescence signals in the body, including tumor tissue injected with ZW800-1, almost disappeared because of the rapid renal excretion behavior of ZW800-1 (Figure 4b). This demonstrates that the MTX moiety of MTX-ZW plays an important role in tumor targeting so that MTX-ZW can be utilized for PTT applications. Moreover, the biodistribution of MTX-ZW was confirmed by comparing the fluorescence signals of major organs collected from mice 4 h post-injection (Figure 4c,d). MTX-ZW exhibited moderate fluorescence signals in the liver and kidneys, as well as tumor tissue and high fluorescence, dominantly detected in the bladder, indicating that the zwitterionic moiety of MTX-ZW could contribute to improving distribution and clearance in the body.

### 2.4. In Vitro and In Vivo Photothermal Effects

**Figure 5:**
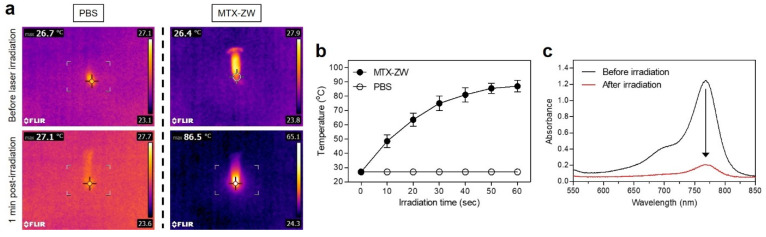
(**a**) In vitro thermal images of the MTX-ZW solution (10 μg/100 μL in PBS; 100 μM concentration is equivalent to a single dose of 0.5 mg/kg) and PBS alone (100 μL) exposed to an 808 nm laser (1.1 W/cm^2^) for 1 min. The maximum temperature was automatically recorded using an infrared thermal camera as a function of the irradiation time. (**b**) Temperature changes in the solutions in each sample were monitored during the 1 min of laser irradiation. (**c**) Photostability of the MTX-ZW solution under laser irradiation. The absorbance changes were measured before and after the 1 min of laser irradiation.

The PTT capabilities of MTX-ZW or PBS solutions in vitro were confirmed by a 1 min exposure to 808 nm laser irradiation (1.1 W/cm^2^). The temperature changes were monitored in real-time using a thermal imager. The temperature of the MTX-ZW solution rapidly increased from 26.4 to 86.5 °C during the 1 min of laser irradiation, while the temperature of PBS alone exhibited almost no change (27.1 °C) under the same conditions (Figure 5a). The temperature of the MTX-ZW solution immediately reached up to ~75 °C during the first 30 s of laser irradiation and continuously increased up to ~86 °C during the next 30 s of irradiation (Figure 5b). This suggests that MTX-ZW has an efficient photothermal conversion capability so that MTX-ZW can be utilized for in vivo PTT applications. Additionally, the absorbance of MTX-ZW was measured before and after the 1 min of laser irradiation in order to determine the photostability of MTX-ZW (Figure 5c). Similar to the previous studies using ZW800-1 and ZW800-AM [4,25], the absorbance of the MTX-ZW solution significantly decreased after 1 min of laser irradiation. This demonstrates that the polymethine structure of NIR fluorophores can be destroyed under NIR light irradiation after showing light-to-heat conversion.

**Figure 6:**
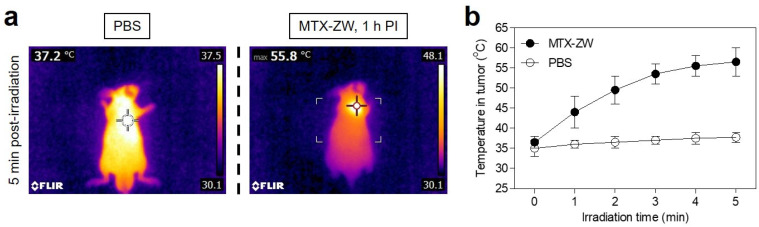
(**a**) Whole-body thermal images of tumor-bearing mice 1 h after injection of PBS or MTX-ZW upon exposure to 808 nm laser irradiation (1.1 W/cm^2^) for 5 min. (**b**) Temperature changes at the tumor sites in each treatment group were monitored during the 5 min of 808 nm laser irradiation. Images are representative of 3 mice per treatment group.

After confirming the superior photothermal conversion performance in vitro, the PTT capability study of MTX-ZW in vivo was conducted using an HT-29 tumor-bearing mouse model under NIR laser irradiation. MTX-ZW or PBS were injected intravenously into the HT-29 xenograft mouse model 1 h before laser irradiation. Subsequently, tumor sites were exposed to 808 nm laser irradiation at 1.1 W/cm^2^ for 5 min. The tumor temperature injected with MTX-ZW immediately increased up to ~56 °C during the 5 min of laser irradiation, whereas the tumor temperature treated with PBS showed almost no change (37.2 °C) under the same condition (Figure 6a). Similar to the previous PTT studies using ZW800-1 and ZW800-AM [4,25], the MTX-ZW-treated tumors began to show that the therapeutic temperature reached around 50 °C at 2 min post-irradiation. Furthermore, the tumor temperatures steadily increased up to ~56 °C for the next 3 min of laser irradiation (Figure 6b). This demonstrates that the MTX-ZW conjugate is sufficient to use for effective photothermal cancer therapy as a rapid tumor-targetable PTT agent.

### 2.5. In Vivo Photothermal Therapeutic Efficacy

**Figure 7:**
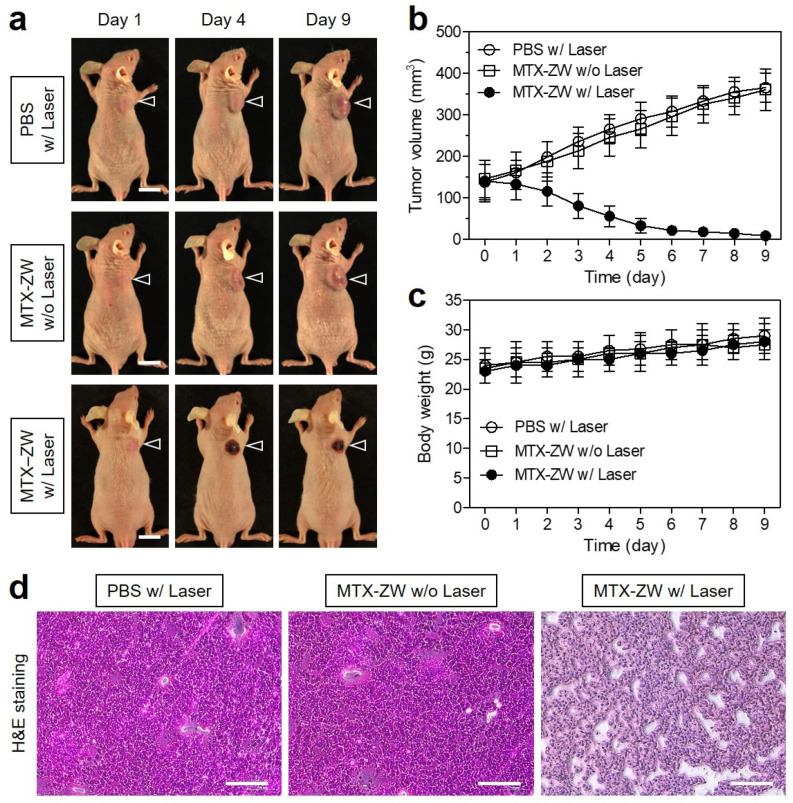
In vivo NIR phototherapeutic efficacy. (**a**) Representative photos of tumor size changes in HT-29 tumor-bearing mice for 9 days after different treatments. The laser groups were treated 1 h post-injection of PBS or MTX-ZW, followed by 808 nm laser irradiation (1.1 W/cm^2^) for 5 min. The tumor sites are indicated by arrowheads. Scale bars = 1 cm. Images are representative of 3 mice per treatment group. (**b**) Tumor growth rates and (**c**) body weights of each treatment group were monitored for 9 days. (**d**) Tumor sections stained with H&E from each group after 24 h of different treatments. Scale bars = 100 μm.

For the final step, the tumors in each treatment group were monitored for 9 days to evaluate the phototherapeutic effect of MTX-ZW (Figure 7a,b). The tumors treated with PBS followed by laser irradiation displayed similar growth patterns to that of the tumors injected with MTX-ZW without laser treatment because of no therapeutic effect induced by laser irradiation alone and the short tumor retention time of MTX-ZW, respectively. The mice group treated with MTX-ZW and laser irradiation revealed highly effective suppression of tumor volume, indicating that the combination of MTX-ZW and NIR laser irradiation can successfully eliminate the tumor without recurrence during the course of treatment. Additionally, no distinct change in body weight was observed in the MTX-ZW treatment groups during the treatment period, which demonstrated a favorable tolerance of the PTT and good in vivo biocompatibility of MTX-ZW (Figure 7c). Finally, the tumors collected from each group 24 h after different treatments were stained with H&E to reconfirm the histological findings of the PTT effect (Figure 7d). Complete necrosis was observed in the entire tumor treated with MTX-ZW and laser irradiation, while no change in the cancer cells was confirmed in the treatment groups using PBS with laser irradiation and MTX-ZW alone without laser irradiation. This demonstrates that MTX-ZW generated a mean tumor temperature of ~56 °C that was quite sufficient to induce cell apoptosis and necrosis.

## 3. Discussion

Previously, many studies have focused on improving the therapeutic effect of anticancer drugs in cancer therapy by using macromolecular drug carriers designed with various kinds of organic or inorganic macromolecules such as proteins, antibodies, and mainly synthetic polymers [27]. In addition, plasmonic nanomaterials (e.g., gold, silver, and copper) have been extensively used as nanocarriers or contrast agents due to their strong light absorption, high photothermal properties, and excellent photostability, which could be combined with drug delivery and phototherapy [28]. In general, this approach has been mostly successful with certain advantages, including enhanced drug stability, drug loading, and targeting efficiency. While in vivo drug delivery and drug potency of the polymer-drug complexes are substantially improved, the ongoing issues of complicated synthetic processes and unsolved biological safety still remain key challenges to future clinical applications [29]. In an attempt to reduce the nonspecific tissue/organ uptake of nanoparticles through the control of particle size and surface charge [30], there has been a need to simplify the preparation process and optimize the key physicochemical properties for the development of ideal therapeutic agents. Alternatively, it is particularly appealing to create multifunctional NIR fluorophores utilized in conjugation with anticancer drugs to enable tumor targeting for image-guided treatment, photosensitivity for laser-induced PTT, and rapid excretion for reducing systemic toxicity.

To improve the use of anticancer drugs for tumor-targeted phototherapeutic applications, the conjugated NIR fluorophore should be designed to be very soluble in aqueous solutions and can be rapidly eliminated from the body after a systemic administration over a certain period of time. In this regard, ZW800-1 is well-known as a highly water-soluble zwitterionic NIR fluorophore with a geometrically-balanced, electrically-neutral surface charge, which leads to rapid renal clearance and ultralow nonspecific background uptake [11]. Moreover, a reactive functionality of the ZW800-1 structure can be modified as either a free amino group or carboxylic acid moiety for further bioconjugation with various small molecule drugs. Recently, our group reported a rapid renal-clearable conjugate by combining ZW800-1 with isoniazid as an antibiotic drug to improve tumor uptake for use in effective cancer PTT [4]. To conjugate with the MTX in this study, the ZW800-1 structure was functionalized with an amino group through tyramine linker substitution on the heptamethine bridge. Then, the poorly water-soluble MTX can be utilized for efficient photothermal cancer treatment with improved tumor imaging and rapid body clearance, owing to the excellent optical and physicochemical properties and in vivo performance of the covalently conjugated zwitterionic NIR fluorophore.

The most important feature of MTX is to induce an inhibition effect on the synthesis of cellular DNAs, thereby leading to cell apoptosis [22]. Although the MTX-ZW conjugate could be accumulated at the tumor tissue with a significantly higher fluorescence intensity than at the normal tissue adjacent to the tumor, the sufficient tumor accumulation for efficient use of photothermal energy was maintained only up to 4 h post-injection. In this regard, the MTX-ZW conjugate was incapable of regulating MTX activity to show chemotherapeutic effects on tumor cells because of its short retention time in tumor tissue. Considering the advantages of the MTX-ZW conjugate, however, MTX could serve as a tumor-targeting ligand for designing various small molecule drug conjugates for diagnostic and phototherapeutic applications.

This study is the first attempt to systematically evaluate the phototherapeutic efficacy of a newly developed MTX-ZW conjugate in vitro and in vivo. We suggest several important aspects of this conjugate: (i) MTX-ZW shows good water solubility due to the highly water-soluble zwitterionic fluorophore; (ii) MTX-ZW is rapidly eliminated from the body within 4 h of injection due to the surface charge-balanced zwitterionic fluorophore; (iii) MTX-ZW exhibits preferential tumor uptake due to the folate antagonist MTX; (iv) MTX-ZW revealed fast tumor targeting but short retention time in the tumor only up to 4 h post-injection, affected by the unique in vivo performance of the zwitterionic fluorophore; (v) MTX-ZW is capable of generating photothermal energy under NIR laser irradiation to kill cancer cells, due to the strong NIR light-absorbing zwitterionic fluorophore. Those results indicate that MTX-ZW could be performed as a bifunctional PTT agent armed with high tumor targetability of MTX and excellent in vivo performance and photosensitivity of zwitterionic NIR fluorophore.

Still, there remain several issues to overcome in future studies. First, the prolonged retention time of MTX-ZW in the tumor requires to play not only a key role in tumor targeting but also a crucial role in the intrinsic anticancer activity of the MTX moiety. Thus, macromolecular drug carriers might be used for the longer retention time of MTX-ZW in the tumor, thereby enabling synergistic chemotherapy and phototherapy. Second, the MTX-ZW conjugate can be redesigned by using the disulfide bridge as a cleavable linker rather than amide bond formation to evaluate the chemotherapeutic efficacy of the MTX moiety for the process of prodrug activation. However, PTT may not be applicable in this case. Third, in vitro and in vivo experiments of MTX-ZW were performed only with HT-29 cells in the present study. Further analyses are required to confirm those effects on animal models for different cancer types.

## 4. Materials and Methods

### 4.1. Conjugation of Methotrexate to the Zwitterionic NIR Fluorophore (MTX-ZW)

All reagents and solvents were purchased from Sigma-Aldrich (St. Louis, MO, USA) and were used without further purification. The ZW800-Cl heptamethine cyanine fluorophore was prepared as described previously [13]. Before introducing a tyramine linkage on the meso-chlorine atom of ZW800-Cl, tert-butyloxycarbonyl (Boc)-protected tyramine was prepared by adding triethylamine (TEA; 0.23 g, 2.28 mmol) and Boc anhydride (0.5 g, 2.29 mmol) into tyramine solution (0.21 g, 1.53 mmol) in dimethylformamide (DMF; 5 mL). The reaction mixture was stirred at ambient temperature for 2 h. To the above solution, under nitrogen atmosphere, sodium hydride (0.04 g, 1.6 mmol) was added, and the mixture was stirred at ambient temperature for 1 h. Subsequently, ZW800-Cl (0.1 g, 0.12 mmol) was added to the above solution, and the mixture was stirred at ambient temperature for 17 h. For the Boc deprotection, a solution of trifluoroacetic acid (TFA) and water (5 mL, 50/50 *v*/*v*%) was mixed with the above solution and stirred at ambient temperature for an additional 2 h. The crude mixture was crystallized with ethyl acetate, collected, and dried under vacuum. The crude product was separated using a preparative high-performance liquid chromatography (HPLC) system (Waters, Milford, MA, USA) to obtain the amine-functionalized zwitterionic NIR fluorophore (ZW800-AM). Finally, methotrexate (10 mg, 0.02 mmol) was conjugated to ZW800-AM (10 mg, 0.01 mmol) in the presence of 4-(4,6-dimethoxy-1,3,5-triazin-2-yl)-4-methylmorpholinium chloride (DMT-MM; 5 mg, 0.02 mmol) in dimethyl sulfoxide (DMSO; 5 mL) at ambient temperature for 12 h. The crude product was repeatedly separated using a preparative HPLC system to remove unreacted products and DMSO, followed by freeze-drying to obtain a purified solid powder. The molecular weights of the purified ZW800-AM and MTX-ZW were confirmed by Dionex UltiMateTM 3000 mass spectrometry system (Thermo Scientific, Waltham, MA, USA).

### 4.2. Optical and Physicochemical Property Analyses

Optical properties of MTX-ZW were measured in phosphate-buffered saline (PBS, pH 7.4). The absorption spectrum of MTX-ZW was measured using a fiber optic UV-Vis-NIR (200–1025 nm) spectrometer (Ocean Optics, Dunedin, FL, USA). The molar extinction coefficient (*ε*) was calculated using the Beer–Lambert equation. The fluorescence emission spectrum of MTX-ZW was recorded using a SPARK^®^ 10M microplate reader (Tecan, Männedorf, Switzerland) at an excitation wavelength of 710 nm and emission wavelengths ranging from 770 to 900 nm. In silico predictions of the distribution coefficient (log*D* at pH 7.4) and topological polar surface area (TPSA) were performed using Marvin and JChem calculator plugins (ChemAxon, Budapest, Hungary).

### 4.3. In Vitro Cell Binding and NIR Fluorescence Microscopy

The human colorectal adenocarcinoma cell line HT-29 was obtained from the American Type Culture Collection (ATCC, Manassas, VA, USA). Cancer cells were maintained in Roswell Park Memorial Institute (RPMI) 1640 medium (Gibco BRL, Paisley, UK) supplemented with a 10% fetal bovine serum (FBS, Gibco BRL) and an antibiotic-antimycotic solution (Welgene, Daegu, South Korea) in a humidified 5% CO_2_ atmosphere at 37 °C. When the cells reached a confluence of approximately 50%, they were rinsed twice with PBS, and the MTX-ZW was added to each well at various concentrations in the range of 2–20 μM. The HT-29 cells were incubated for 24 h at 37 °C and then gently washed with PBS. NIR fluorescence imaging was performed using a Nikon Eclipse Ti-U inverted microscope system (Nikon, Seoul, South Korea). Image acquisition and analysis were performed using the NIS-Elements Basic Research software (Nikon).

### 4.4. In Vitro Cytotoxicity Assay

Cell toxicity and proliferation were evaluated using a 3-(4,5-dimethylthiazol-2-yl)-2,5-diphenyltetrazolium bromide (MTT, Sigma-Aldrich) assay. The HT-29 cells were seeded onto 96-well plates (1 × 10^4^ cells per well). To evaluate the cytotoxicity depending on the MTX-ZW concentration, the cancer cells were treated with the MTX-ZW conjugate (2, 5, 10, 20, and 50 μM) for 1 h and cultured for 24 h after treatment. At each time point, the incubation cell medium was replaced with 100 μL of fresh medium, and 10 μL of the MTT solution was directly added to each 100 μL well. Subsequently, the plates were then incubated for 4 h at 37 °C in a humidified 5% CO_2_ incubator. Finally, the plates were placed in a microplate reader (SPARK^®^ 10M, Tecan) to measure the absorption intensity at 570 nm. Cell viability was calculated using the following formula: cell viability (%) = (*A*_sample_ − *A*_blank_)/(*A*_control_ − *A*_blank_) × 100, where *A* is the average absorbance.

### 4.5. HT-29 Xenograft Mouse Model

Animal studies were performed in accordance with the guidelines approved by the Chonnam National University Animal Research Committee (CNU IACUC-H-2020-19). Adult (6-week-old, ≈25 g) male athymic nude mice were purchased from OrientBio (Gwangju, South Korea). HT-29 cancer cells were cultured and suspended in 100 μL of PBS before being subcutaneously inoculated in the right flank of each mouse (1 × 10^6^ cells per mouse). When tumor sizes reached about 1 cm in diameter between 8 to 10 days post-inoculation, the MTX-ZW solution (10 μg/100 μL in PBS; 100 μM) was administered intravenously. Animals were euthanized for in vivo NIR fluorescence imaging within a designated period of time.

### 4.6. In Vivo Biodistribution and Tumor Imaging

In vivo NIR fluorescence imaging was performed using an FOBI imaging system (NeoScience, Suwon, South Korea). Mice (3 mice per treatment group) were sacrificed 4 h after injection, and their main organs (heart, lungs, liver, pancreas, spleen, kidneys, duodenum, and intestine) were harvested and imaged to confirm the time-dependent biodistribution of MTX-ZW. The fluorescence intensities of the tumors and excised organs were analyzed using ImageJ software (National Institutes of Health, Bethesda, MD, USA).

### 4.7. In Vivo Photothermal Therapeutic Efficacy

MTX-ZW or PBS were intravenously injected into the HT-29 tumor-bearing mice (3 mice per treatment group), and the mice were anesthetized after 1 h. The tumors were treated with a laser (1.1 W/cm^2^, *λ* = 808 nm) for 5 min. Temperature changes at the tumor sites were monitored using a thermal imager (FLIR Systems, Wilsonville, OR, USA). Tumors were excised from the treated mice 24 h after irradiation for subsequent analysis of histological samples stained with hematoxylin and eosin (H&E). To assess the in vivo antitumor effect, the macroscopic tumor growth of each group was observed for 9 days. The tumor volume (V) was measured by the following formula: V = 0.5 × longest diameter × (shortest diameter)^2^.

### 4.8. Statistical Analysis

Statistical analysis was performed by one-way analysis of variance for multiple comparison tests. The results were represented as mean ± standard deviation (S.D.). A value of *p* < 0.05 was considered statistically significant. Curve fitting was performed using the Prism software (GraphPad, San Diego, CA, USA).

### 4.9. Histological Analysis

Resected tumors were preserved for H&E staining and microscopic observation. The tumors were fixed in 4% paraformaldehyde and flash-frozen in an optimal cutting temperature (OCT) compound using liquid nitrogen. Frozen samples were cryosectioned (10 µm thick), stained with H&E, and observed using a microscope. Histological analysis was performed on a Nikon Eclipse Ti-U inverted microscope system (Nikon).

## Data Availability

Not applicable.
